# The Combination of Immunomagnetic Bead-Based Cell Isolation and Optically Induced Dielectrophoresis (ODEP)-Based Microfluidic Device for the Negative Selection-Based Isolation of Circulating Tumor Cells (CTCs)

**DOI:** 10.3389/fbioe.2020.00921

**Published:** 2020-08-06

**Authors:** Po-Yu Chu, Chia-Hsun Hsieh, Min-Hsien Wu

**Affiliations:** ^1^Ph.D. Program in Biomedical Engineering, Chang Gung University, Taoyuan City, Taiwan; ^2^Division of Haematology-Oncology, Department of Internal Medicine, Chang Gung Memorial Hospital (Linkou), Taoyuan City, Taiwan; ^3^Division of Haematology-Oncology, Department of Internal Medicine, New Taipei Municipal TuCheng Hospital, New Taipei City, Taiwan; ^4^College of Medicine, Chang Gung University, Taoyuan City, Taiwan; ^5^Graduate Institute of Biomedical Engineering, Chang Gung University, Taoyuan City, Taiwan; ^6^Department of Chemical Engineering, Ming Chi University of Technology, New Taipei City, Taiwan

**Keywords:** circulating tumor cells, optically-induced dielectrophoresis, microfluidic technology, immunomagnetic microbeads, cell isolation

## Abstract

Negative selection-based circulating tumor cell (CTC) isolation is able to harvest viable, label-free, and clinically meaningful CTCs from the cancer patients’ blood. Nevertheless, its main shortcoming is its inability to isolate high-purity CTCs, restricting subsequent CTC-related analysis. To address this issue, this study proposed a two-step optically-induced dielectrophoresis (ODEP) cell manipulation to process the cell sample harvested by negative selection-/immunomagnetic microbeads-based CTC isolation. The working mechanism is that the ODEP force acting on the cells with and without magnetic microbeads binding is different. Accordingly, the use of ODEP cell manipulation in a microfluidic system was designed to first separate and then isolate the cancer cells from other magnetic microbead-bound cells. Immunofluorescent microscopic observation and ODEP cell manipulation were then performed to refine the purity of the cancer cells. In this study, the optimum operating conditions for effective cell isolation were determined experimentally. The results revealed that the presented method was able to further refine the purity of cancer cell in the sample obtained after negative selection-based CTC isolation with high cell purity (81.6~86.1%). Overall, this study proposed the combination of immunomagnetic bead-based cell isolation and ODEP cell manipulation for the negative selection-based isolation of CTCs.

## Introduction

Metastasis is the main cause of cancer morbidity and mortality (Mehlen and Puisieux, 2006). Circulating tumor cells (CTCs) are those cells that have escaped from the primary tumor tissue into the vasculature and are subsequently present in the blood circulation or lymphatic system (Plaks et al., 2013). The presence of CTCs in the blood circulation is closely associated with cancer metastasis (Plaks et al., 2013). As a result, the fundamental study of CTCs has immense potential for revealing the mechanism behind metastasis. Such a discovery could help scientists develop novel therapeutic methods for cancer treatments. Moreover, in terms of clinical utility, the CTCs obtained from cancer patients’ blood can act as a kind of liquid tumor biopsy, which are useful for various clinical applications [e.g., the selection of personalized therapeutic regimens (Yue et al., 2018), for the evaluation of therapeutic response (Otsuka et al., 2013), or for the detection of cancer disease status (Tsai et al., 2016)].

To achieve these goals, the isolation and purification of high-purity, label-free, viable, and, more importantly, clinically meaningful CTCs from blood samples of cancer patients is crucial. Nevertheless, CTCs are normally rare in a blood sample (Allan and Keeney, 2010), making them technically difficult to isolate and purify. CTCs can be separated and then isolated from the blood samples of cancer patients through physical- or biochemical-based working mechanisms. It is well accepted that physical-based approaches are simple, low-cost, and label-free to operate. Nevertheless, their performance for target cell isolation (e.g., the cell purity achieved: 0.1~10%; (Hosokawa et al., 2010; Hou et al., 2013; Hao et al., 2018) is commonly inferior to the other biochemical methods (Hosokawa et al., 2010; Hou et al., 2013; Hao et al., 2018).

In contrary, the methods based on biochemical mechanism are the mainstream choice in present CTC isolation practices (Joosse et al., 2015). In these methods, immunomagnetic microbead-based cell separation and isolation is the commonly adopted technique for such tasks. Briefly, magnetic microbeads coupled with CTC surface antigen [e.g., mainly epithelial cell adhesion molecule (EpCAM)]-specific antibodies are used to recognize and then selectively bind with CTCs in a cell suspension sample. The magnetic microbead-bound CTCs are subsequently separated from the background blood cells through the exertion of a magnetic field. The above-mentioned CTC isolation process is called the positive selection of CTCs [e.g., the cell purity achieved: 7~51%; Talasaz et al., 2009; Kang et al., 2019). Although the current physical or biochemical CTC isolation protocols have been demonstrated to be workable to isolate CTCs, the existence of background blood cells in the harvested sample remains a problem. This problem could in turn complicate the following analytical works (Chiu et al., 2016; Cho et al., 2018; Kang et al., 2019).

Except for the technical hurdle mentioned above, the present CTC isolation and purification methods might not be able to harvest clinically suitable and label-free CTCs for subsequent applications. First, the concept that CTCs with metastatic potential might experience a transformation called epithelial-to-mesenchymal transition (EMT) has been thoroughly vetted (Mikolajczyk et al., 2011; Gabriel et al., 2016; Swennenhuis et al., 2016). After this transformation, the expression of EpCAM on the cellular surfaces of CTCs is downregulated (Mikolajczyk et al., 2011; Gabriel et al., 2016; Swennenhuis et al., 2016). Owing to this phenomenon, the conventional positive selection-based CTC isolation strategy might not be able to isolate these clinically meaningful CTCs closely associated with cancer metastasis (Lustberg et al., 2014; Peters et al., 2016; Liao et al., 2018; Kang et al., 2019; Nicolazzo et al., 2019). Second, the CTCs obtained from the positive selection-based cell isolation protocol are normally labeled with magnetic microbeads, which could, for example, limit the use of the harvested cells for subsequent CTC culture-based assays. To solve out the technical issues, more recently, the idea of negative selection-based CTC isolation was presented. In operations, erythrocytes are first depleted using, for example, a chemical lysis-based method. The leukocytes in the treated sample are then targeted for removal via the technique of immunomagnetic microbeads-based cell separation and isolation (Peters et al., 2016; Liao et al., 2018; Banko et al., 2019; Kang et al., 2019). Based on this strategy, it has been proven that all possible and label-free CTCs in the blood samples of cancer patients can be obtained (Peters et al., 2016; Liao et al., 2018; Banko et al., 2019; Kang et al., 2019). However, its key technical problem is its inability to harvest high-purity target cells (e.g., the CTC purity achieved: 5~10%; Liao et al., 2017; Kang et al., 2019).

Borrowing from the advances of microfluidic technology, several microscale cell manipulation techniques (Talasaz et al., 2009; Hou et al., 2013; Chiu et al., 2016; Liao et al., 2018; Kang et al., 2019) were proposed to be integrated into microfluidic systems for CTC isolation (Cho et al., 2018). Leveraging the characteristic feature of miniaturized scale, overall, these microfluidic systems have been successfully demonstrated to have better performances of CTC isolation compared with conventional CTC isolation schemes (Cho et al., 2018). Among the cell manipulation techniques, optically-induced dielectrophoresis (ODEP)-based techniques particularly attract the biologists’ interest due to their ease of operation. In practice, one can simply use a commercial digital projector to display light images on an ODEP microfluidic system to manipulate biological cells of interest in a simple, flexible, user-friendly, and low-cost manner (Huang et al., 2013; Chiu et al., 2016; Chou et al., 2017; Liao et al., 2018; Chu et al., 2019a). The utilization of ODEP cell manipulation in micro-scale systems has been successfully demonstrated for various biological applications [e.g., the isolation of rare cells (Huang et al., 2013; Chiu et al., 2016; Chou et al., 2017; Liao et al., 2018), the sorting or isolation of cancer cells (Chu et al., 2019a) or bacteria (Wang et al., 2020) with varied drug resistance, or identification of sperms’ viability and motility (Ohta et al., 2010)]. In addition, we have also previously exhibited the use of such a technique in microfluidic systems for the isolation of CTCs in a higher performance manner in comparison with conventional counterparts (Chiu et al., 2016). Nevertheless, the utilization of ODEP cell manipulation for the high-purity and label-free isolation of viable CTCs based on a negative selection strategy has not yet been explored.

In this study, we proposed the combination of immunomagnetic bead-based cell isolation and ODEP-based cell manipulation for the negative selection-based isolation of CTCs. In operations, a conventional negative selection-based CTC isolation process using the standard immunomagnetic microbeads-based cell isolation technique (Chiu et al., 2016; Peters et al., 2016; Liao et al., 2018) was first carried out to isolate CTCs from the blood samples of cancer patients. After that, a two-step ODEP cell manipulation was performed to process the cell sample harvested from the previous cell isolation process to refine the purity of the cancer cells. The first working mechanism is that the ODEP force acting on the magnetic microbead-bound leukocytes, the main cell population in the cell sample obtained from a negative selection-based CTC isolation protocol, and the other cells without magnetic microbeads binding are different. Based on this difference, the use of ODEP cell manipulation in a microfluidic system was designed to first separate and then isolate the cancer cells from the other magnetic microbead-bound leukocytes. The cell sample obtained was soon followed by the second step, during which immunofluorescence microscopic observation and ODEP cell manipulation were carried out to further refine the cell purity of the target cell population. In this study, an ODEP microfluidic system was designed and fabricated. In addition, the optimum ODEP operating conditions [e.g., size (50 nm) and concentration (0.2 mg ml^–1^) of magnetic microbeads, the angle between the rectangular light bar and sample flow direction (15°), and the sample flow rate (1.0 μl min^–1^)] for effective cell separation and isolation were determined experimentally. Finally, the performance of cancer isolation and purification via the presented two-step ODEP cell manipulation process was experimentally assessed. The results revealed that the presented two-step ODEP cell manipulation process was able to further refine the cancer cell purity of the sample obtained after a negative selection-based CTC isolation process with high cell purity (81.6~86.1%). Overall, this study proposes a hybrid method for the negative selection-based isolation of CTCs. In addition to CTC isolation, the presented method is also useful in other research areas in which the isolation of high-purity, label-free, and viable cells is required.

## Materials and Methods

### The ODEP Microfluidic Chip and Operating Setup

**FIGURE 1:**
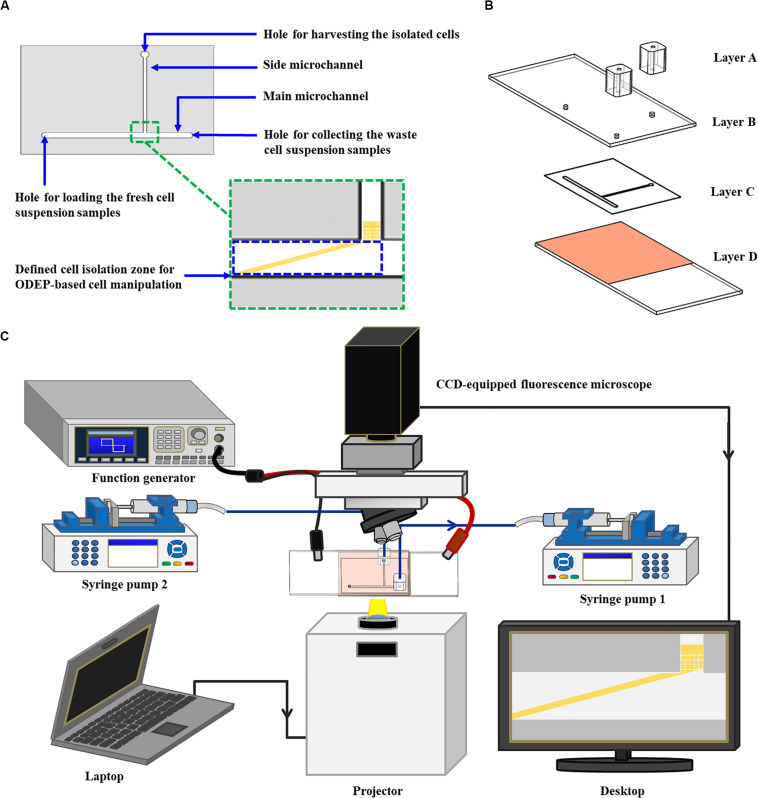
Schematic presentation of the **(A)** top-view design layout and **(B)** the structure of an ODEP microfluidic chip (Layer A: two PDMS adapters for tubing connection; Layer B: an ITO glass; Layer C: a double-sided adhesive tape with a hollow T-shaped microchannel; Layer D: an ITO glass coated with a photoconductive layer), and the **(C)** the whole operating setup.

In this study, a microfluidic chip encompassing a T-shaped microchannel was designed (Figure 1A). The functions of the designed main microchannel (L: 21.0 mm, W: 1.0 mm, H: 50.0 μm) and side microchannel (L: 14.0 mm, W: 500.0 μm, H: 50.0 μm) were for transporting the cell suspension sample and for collecting the isolated cells, respectively. In the design, moreover, the ODEP cell manipulation for the continuous sorting and isolation of cancer cells from the surrounding magnetic microbead-bound leukocytes was first performed at the defined cell isolation zone (L: 4.2 mm, W: 1.0 mm, H: 50.0 μm) of the main microchannel (Figure 1A). The structure of the ODEP microfluidic chip, encompassing two home-made polydimethylsiloxane (PDMS) adapters for tubing connection (Layer A), an indium-tin-oxide (ITO) glass (7 Ω, 0.7 mm; Ritek, TWN) (Layer B), a double-sided adhesive tape (L298, thickness: 50.0 μm, Sun-yieh, TWN) with a hollow T-shaped microchannel (Layer C), and a bottom ITO glass coated with a layer of photoconductive material, is shown in Figure 1B. In the Layer D, the photoconductive material was composed of a 12-nm-thick p-type hydrogenated amorphous silicon layer and a 500-nm-thick hydrogenated amorphous silicon layer. The fabrication processes for each layer (Figure 1B) were based on PDMS replica molding, metal mold-punching fabrication, and thin-film technology, as well described previously (Liao et al., 2018; Chu et al., 2019a, b).

For the following assembly process, the two PDMS adapters were bonded with the ITO glass substrate (Layer B) with the aid of O_2_ plasma surface treatment. This bonding was followed by assembly with the processed ITO glass substrate (Layer D) with the aid of the prepared double-sided adhesive tape (Layer C) (Figure 1B). In terms of the operating setup (Figure 1C), two syringe pumps were used for cell suspension transportation in the designed ODEP microfluidic chip. For the ODEP cell manipulation in the microfluidic chip, the other experimental setup was based on our previous studies (Liao et al., 2018; Chu et al., 2019a, b). Briefly, a function generator was utilized to apply an alternating current (AC) voltage between the two ITO glasses. A digital projector (EB-X05, Epson, JP) connected with a computer was used to project light images onto the photoconductive material. In addition, a CCD-equipped fluorescence microscope (Zoom 160, OPTEM, US) was designed to observe the ODEP cell manipulation process in the microfluidic chip. A photograph of the overall experimental setup is provided as a Supplementary Figuer 1.

### The Working Mechanism for the Separation of Cancer Cells From the Surrounding Magnetic Microbead-Bound Cells via ODEP Cell Manipulation

Cell manipulation using the ODEP mechanism was utilized to sort and then separate the targeted cancer cells from the surrounding magnetic microbead-bound cells in this study. The key working principle is described as follows. In terms of microparticle (e.g., biological cells) manipulation using the ODEP phenomenon (Chu et al., 2019a, b), briefly, an electric voltage is applied between the top and bottom substrates of an ODEP system. This application in turn generates a uniform electric field between the two substrates. When microparticles with dielectric properties are suspended in solution under the generated electric field, charges can be electrically polarized on the microparticles’ surface. When the photosensitive substrate (e.g., Layer D; Figure 1B) of an ODEP system is projected with a light, the light can lead to a voltage fall across the liquid layer within the light-illuminated area and then induces a non-uniform distribution of an electric field between the top and bottom substrates. The interaction between an electrically polarized microparticle and the non-uniform electric field created due to light illumination was employed to manipulate microparticles. Based on the abovementioned phenomenon, overall, one can manipulate the light image projected onto the photosensitive layer of an ODEP system to manageably control a microparticle.

The ODEP force generated on a microparticle can be described by Eq. (1) (Chu et al., 2019a, b):

(1) F=DEP2πrε3ε0Rem[f]CM∇|E|2

In Eq. (1), r, ε_0_, ε_*m*_, ∇| E| ^2^, and Re[f_CM_] represent the microparticle radius, vacuum permittivity, relative permittivity of the surrounding solution, gradient of the applied electrical field squared, and real part of the Clausius–Mossotti factor (f_CM_), respectively. For a given property of the working solution, the direction of the ODEP force generated on a microparticle is determined by Re[f_CM_] (Valley et al., 2008; Hwang and Park, 2011; Chen and Yuan, 2019). Re[f_CM_] is further determined by the frequency of the applied electric voltage as well as the dielectric property of a microparticle (Valley et al., 2008; Hwang and Park, 2011; Chen and Yuan, 2019). If the Re[f_CM_] is positive, the manipulated microparticles are attracted within a light image and the light image can be used to pull the microparticles for manipulation (i.e., positive ODEP force). When Re[f_CM_] is negative, conversely, the manipulated microparticles are repulsed by a light image and are thus located outside of the light image. In this situation, the light image can be used to push the microparticles for manipulation purposes (i.e., negative ODEP force) (Valley et al., 2008; Hwang and Park, 2011; Chen and Yuan, 2019).

According to the abovementioned phenomena, the nature (e.g., positive or negative) of the ODEP force generated on a microparticle is dependent on the dielectric property of the microparticle at a given frequency of electric voltage. For example, microbeads and biological cells exhibited negative and positive ODEP forces, respectively, under specific electric conditions (Chiou et al., 2005; Hwang and Park, 2011; Chen and Yuan, 2019). Based on this fact, it might be possible to fine tune the ODEP force generated on a cell if magnetic microbeads are bound onto it. If this speculation is workable, one can simply use ODEP cell manipulation to sort and separate the cancer cells from the surrounding magnetic microbead-bound cells, in which these cells are originally difficult to sort and separate via ODEP cell manipulation due to their similar size. In this study, this speculation was tested and described in section “The Optimum ODEP Operating Conditions for the Isolation of SW620 Cancer Cells From Magnetic Microbead-Bound Jurkat cells.”

### The Working Mechanism of Using the ODEP Microfluidic System to Isolate and Purify Cancer Cells From Magnetic Microbead-Bound Jurkat Cells

**FIGURE 2:**
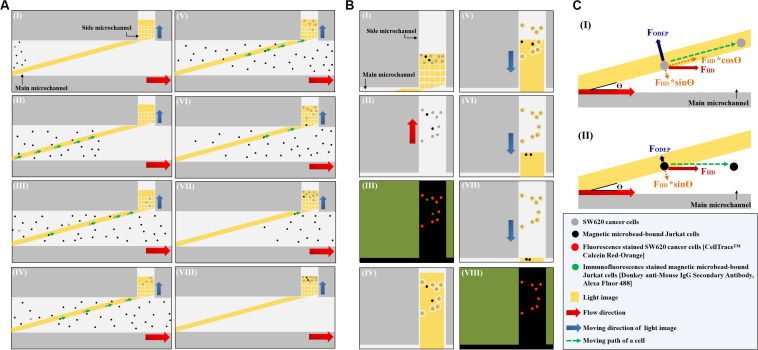
Schematic presentation (top view) of the working mechanism for the continuous isolation and purification of cancer cells from the magnetic microbead-bound Jurkat cells; **(A)** the first step ODEP cell manipulation process: (I): a static rectangular light bar (L: 3.9 mm, W: 100 μm) with a particular angle (e.g., 15°) to the flow direction of the cell suspension was designed at the defined cell isolation zone of the main microchannel, (I–VIII): most of the magnetic microbead-bound Jurkat cells (black dots) in the sample flow were not trapped by the light bar and thus flowed to the downstream of the main microchannel, whereas most of the SW620 cancer cells (gray dots) flowing through the main microchannel were trapped by the light bar and then delivered along the light bar to the side microchannel for cell collection (indicated by arrows), **(B)** the second step of the ODEP cell manipulation process: (I) a certain amount of the cancer cells were collected in the side microchannel, (II) the liquid flow in the side microchannel was driven to flux the cells collected so that they were evenly spread within the side microchannel, (III) fluorescence microscopy observation was carried out to identify the species of the cells [i.e., fluorescence stained SW620 cancer cells (the red dots) and immunofluorescence stained magnetic microbead-bound Jurkat cells (the green dots)] collected in the side microchannel for positioning the SW620 cancer cells, (IV) static circular light images were illuminated on each SW620 cancer cell. Meanwhile, the side microchannel was projected with a rectangular light bar to manipulate the magnetic microbead-bound Jurkat cells into the side microchannel, in which O-ring-like non-illuminated patterns were designed as partitions to separate the light-illuminated SW620 cancer cells and the other cells, (V–VII) the rectangular light bar on the side microchannel was then moved to manipulate the magnetic microbead-bound Jurkat cells to remove them from the side microchannel, and this process was repeated for 5 times, (VIII) through the previous process, the cell purity of the SW620 cancer cells in the side microchannel was greatly improved, **(C)** the working mechanism of using a static rectangular light bar both as a virtual cell filter and a virtual cell track for continuous cancer cell separation and isolation: (I) when the F_ODEP_ (ODEP manipulation force) acting on a cell is greater than the component force of F_HD_ (i.e., F_HD_^∗^sinΘ, Θ: the angle between the rectangular light bar and the flow direction of the cell suspension), the cell could move along the rectangular light bar, driven by another component force of F_HD_ (i.e., F_HD_^∗^cosΘ), (II) when the F_ODEP_ is less than F_HD_^∗^sinΘ, conversely, a moving cell might not be trapped by the designed light bar image and thus flows through the light image area.

In this study, the ODEP microfluidic system (Figure 1C) was utilized to process the cell sample obtained from the negative selection-based CTC isolation scheme using the standard immunomagnetic microbead-based cell isolation technique. In operations, a two-step ODEP cell manipulation process, schematically presented in Figures 2A,B, was designed to sort, separate, and then isolate SW620 cancer cells (a cell line used as model CTCs) from magnetic microbead-bound Jurkat cells (a cell line used as model leukocytes). In this work, a static rectangular light bar (L: 3.9 mm, W: 100.0 μm) with a particular angle (e.g., 15°) to the flow direction of the cell suspension was designed at the defined cell isolation zone of the main microchannel (Figure 2AI). In the design, the static rectangular light bar not only functioned as a virtual cell filter that sorted and separated the cells with and without magnetic microbead binding but also worked as a virtual track that continuously guided the cells without magnetic microbeads binding to the side microchannel for collection. As illustrated in Figures 2AI–VII, most of the magnetic microbead-bound Jurkat cells in the sample flow were not trapped by the rectangular light bar and thus flowed downstream of the main microchannel for waste sample collection. For the SW620 cancer cells flowing through the main microchannel, conversely, most of them were trapped by the rectangular light bar and then delivered along the light bar to the side microchannel for cell collection (Figures 2AI–VIII), indicated by arrows). After a certain amount of the cancer cells were collected in the side microchannel, the second step of the ODEP cell manipulation (Figure 2B) was performed for the further purification of cancer cells. First, the liquid flow (0.1 μl min^–1^) in the side microchannel was driven for 15~20 s to flux the cells collected so that they were evenly spread within the side microchannel, facilitating the following cell manipulation using ODEP mechanism (Figures 2BI,II). After that, fluorescence microscopy observation was carried out to identify the species of the cells collected in the side microchannel (Figure 2BIII). Through this process, SW620 cancer cells (red dots) were then positioned. This positioning was followed by projecting static circular light images on each SW620 cancer cell (the red dot) to generate an ODEP force for anchoring them on the bottom surface of the side microchannel (Figure 2BIV). Meanwhile, the side microchannel was projecting with a rectangular light bar (L: 1.2 mm; W: 420.0 μm) to manipulate the magnetic microbead-bound Jurkat cells (green dots) into the side microchannel, in which O-ring-like non-illuminated patterns were designed as partitions to separate the light-illuminated SW620 cancer cells and the outside part, as illustrated in Figure 2BIV. The rectangular light bar on the side microchannel was then moved (10 μm s^–1^) to manipulate the magnetic microbead-bound Jurkat cells to remove them from the side microchannel, leaving high-purity SW620 cancer cells in the side microchannel (Figures 2BV–VII). The above process was repeated for 5 times so as to obtain high-purity SW620 cancer cells. After the purification of the SW620 cancer cells in the side microchannel (Figure 2BVIII), the isolated SW620 cancer cells were then obtained via the through- port connecting the tubing and a suction-type syringe pump 2, as illustrated in Figure 1C.

In this study, as mentioned earlier, a static rectangular light bar serving both as a virtual cell filter and a virtual cell track was designed at the defined cell isolation zone of the main microchannel (Figure 2AI). Its working mechanism is described herein (Figure 2C). When a cell is in the main microchannel, it flows in accordance with the liquid flow direction due to hydrodynamic force (F_HD_) (Figure 2CI). When the cell flows through the rectangular light bar area, its movement could be altered due to the ODEP force (F_ODEP_) acting on it. At a given electric condition (e.g., 10 Vpp and 3 MHz) (Chen and Yuan, 2019; Chu et al., 2019b), a cell could experience positive ODEP force under an ODEP field. In this situation, the cell is attracted to the edge of a light image (Hwang and Park, 2011; Chou et al., 2017; Chen and Yuan, 2019; Chu et al., 2019b) and the F_ODEP_ exerted on such a cell is perpendicular to the edge of the light image (Hu et al., 2005; Chou et al., 2017), as shown in Figure 2CI. When the movement alteration of the cell is observed (e.g., it moves along the rectangular light bar image), it might indicate that the F_ODEP_ is greater than the component force of F_HD_ (i.e., F_HD_^∗^sinΘ, Θ: the angle between the rectangular light bar and the flow direction of the cell suspension). Under this circumstance, the cell could move along the rectangular light bar, driven by another component force of F_HD_ (i.e., F_HD_^∗^cosΘ) as illustrated in Figure 2CI. When the F_ODEP_ is less than F_HD_^∗^sinΘ, conversely, a moving cell might not be trapped by the designed light bar image and thus flows through the light image area, as shown in Figure 2CII. Because the ODEP force acting on the SW620 cancer cell is expected to be greater than that on a magnetic microbead-bound Jurkat cell, the former could be trapped and then flow along the rectangular light bar (i.e., Figure 2CI) to the side microchannel for collection, whereas the latter could directly flow through the light image area (i.e., Figure 2CII). Based on this phenomenon, SW620 cancer cells can be sorted, separated, and then isolated from magnetic microbead-bound Jurkat cells in a continuous flow manner.

### The Optimum ODEP Operating Conditions for the Isolation of SW620 Cancer Cells From Magnetic Microbead-Bound Jurkat Cells

In the presented ODEP microfluidic system, ODEP cell manipulation was utilized for the continuous separation and isolation of the desired cancer cells from the surrounding magnetic microbead-bound Jurkat cells, as schematically presented in Figure 2A. In the ODEP setting, the electric voltage and frequency were set at 10 Vpp and 3 MHz, respectively (Chu et al., 2019b). In addition, the ODEP manipulation force (Chou et al., 2017; Chu et al., 2019a, b), a net force between the ODEP force and friction force, acting on the manipulated cells (e.g., the magnetic microbead-bound Jurkat cells or SW620 cancer cells) was then experimentally evaluated based on the method described previously (Chou et al., 2017; Chu et al., 2019a, b). In a steady state, the ODEP manipulation force acting on a cell is balanced by the viscous drag of fluid acting on such a cell under continuous flow condition. As a result, the hydrodynamic drag force of a moving cell was used to evaluate the net ODEP manipulation force of a cell according to Stokes’ law (Eq. 2) (Chou et al., 2017; Chu et al., 2019a, b):

(2) F=6⁢π⁢r⁢η⁢v

In Eq. (2), *r*,η, and *v* denote the cellular radius, the fluidic viscosity of the fluid, and the velocity of a moving cell, respectively. Based on Stokes’ law, therefore, the ODEP manipulation force acting on the cells tested can then be experimentally evaluated through measurements of the maximum velocity of a moving light image that can manipulate these cells (Chou et al., 2017; Chu et al., 2019a, b).

To test the speculation described in section “The Working Mechanism for the Separation of Cancer Cells From the Surrounding Magnetic Microbead-Bound Cells via ODEP Cell Manipulation,” Jurkat cells were bound with streptavidin-coated magnetic microbeads of different sizes [diameter: 2 μm (11205D, Invitrogen, US), 1 μm (65001, Invitrogen, US), and 50 nm (SV0050, Ocean Nanotech, US), respectively] and different concentrations (e.g., 0.1, 0.2, and 0.4 mg ml^–1^) via aid of a biotin-coated anti-human CD45 antibody (Mouse IgG1, tcta30459, Taiclone Biotech Corp., TWN). The ODEP manipulation force generated on the magnetic microbead-bound Jurkat cells and SW620 cancer cells was then evaluated experimentally based on the abovementioned method. The treatment conditions (i.e., the size and concentration of the magnetic microbeads) that led to a significant difference in the ODEP manipulation force between the magnetic microbead-bound Jurkat cells and SW620 cancer cells were then selected for the following tests. Based on the selected operating conditions of magnetic microbeads, they were further tested in the negative selection-based cancer cell isolation process (Chiu et al., 2016; Liao et al., 2017, 2018; Kang et al., 2019) using the standard immunomagnetic microbeads-based cell isolation technique. In the tests, briefly, streptavidin-coated magnetic microbeads with the operating conditions selected were designed to selectively bind with Jurkat cells via the aid of biotin-coated anti-human CD45 antibodies. Briefly, Jurkat cells and biotin-coated anti-human CD45 antibodies (concentration: 2.5 μg ml^–1^ per 10^6^ cells) were mixed and then incubated in phosphate-buffered saline (PBS) with 2% fetal bovine serum (FBS) and 1 mM ethylenediaminetetraacetic acid (EDTA) at 4°C for 10 min. After incubation, the sample was washed twice using PBS with 2% FBS and 1 mM EDTA to remove any unbound antibodies. After that, the Jurkat cells bound with biotin-coated anti-human CD45 antibodies were mixed and incubated with the abovementioned streptavidin-coated magnetic microbeads at 4°C for 1 h. In the following step, most of the magnetic microbead-bound Jurkat cells (e.g., ~99%; Wu et al., 2014; Liao et al., 2017; Kang et al., 2019) were expected to be removed due to the exertion of a magnetic field (EasySep^TM^ Magnet, StemCell Technologies, CAN), leaving few of them in the treated cell sample. Based on the aforementioned evaluation method of ODEP manipulation force, the ODEP manipulation force of the magnetic microbead-bound Jurkat cells remaining in the treated cell sample was then experimentally evaluated. The purpose was to explore whether it was still significantly different from that of SW620 cancer cells, as showed in previous tests. Based on this evaluation, the final operation condition of streptavidin-coated magnetic microbeads in terms of their size and concentration was then determined for subsequent works.

As described earlier (Figure 2A), furthermore, the static rectangular light bar functioning as a virtual cell filter was designed in the cell isolation zone of the main microchannel to sort and separate the cells with and without magnetic microbead binding. To determine the optimal angle (between the rectangular light bar and the flow direction of the cell suspension) capable of achieving better cell separation performance, the following evaluation was carried out. Briefly, the cell trapping percentage (%) of SW620 cancer cells and magnetic microbead-bound Jurkat cells trapped at the rectangular light bar area was experimentally evaluated under different angles (15°, 30°, 45°, 60°, 75°, and 90°) and flow rates (0.5, 1.0, and 1.5 μl min^–1^). Due to the technical limitation (i.e., experimental setup), the minimum angle of the rectangular light bar to the flow direction was 15^o^, under which the rectangular light bar can fully cross the main microchannel within the ODEP working area (4.2 mm ^∗^ 3.1 mm; L^∗^W) (Figure 5AI). In this work, the cell trapping percentage (%) was defined as the number of cells trapped at the light image area over the total cell number (i.e., the number of cells trapped at the light image area + the cells collected in the waste).

## Performance Evaluation of Cancer Cell Isolation Using Two-Step Odep Cell Manipulation After a Negative Selection/Immunomagnetic Microbead-Based Cancer Cell Isolation Process

To test whether the proposed method (Figure 2) can refine the cell purity of the cancer cells isolated from a negative selection-based CTC isolation scheme, the following experimental work was carried out. In this study, Jurkat cells and SW620 cancer cells were used as the model cells of leukocytes and CTCs, respectively. To mimic the cell sample obtained after a conventional negative selection-based CTC isolation scheme (Chiu et al., 2016; Liao et al., 2017, 2018; Kang et al., 2019), as described in section “The Optimum ODEP Operating Conditions for the Isolation of SW620 Cancer Cells From Magnetic Microbead-Bound Jurkat Cells,” streptavidin-coated magnetic microbeads were designed to selectively bind with Jurkat cells via the aid of biotin-coated anti-human CD45 antibodies. After removing most of the magnetic microbead-bound Jurkat cells via the exertion of a magnetic field, the cells remaining in the sample were collected for immunofluorescent staining using Donkey anti-Mouse IgG Secondary Antibody, Alexa Fluor 488 (A-21202, Invitrogen, US) (i.e., green fluorescent images). After that, the fluorescence stained SW620 cancer cells (CellTrace^TM^ Calcein Red-Orange, C34851, Invitrogen, US) (i.e., red-orange fluorescent images) with different levels were spiked in the cell sample mentioned above to form the cell samples containing 5 and 10% SW620 cancer cells. The purpose of this cell sample preparation was to mimic the cell purity [e.g., 5~10% (Liao et al., 2017; Kang et al., 2019)] of CTCs in the cell samples normally obtained from a conventional negative selection-based CTC isolation scheme. It was followed by replacing the background solution of cell samples with a sucrose buffer (270~290 mOsmol kg^–1^ and 1~5 μS cm^–1^) before the subsequent ODEP cell manipulation. The prepared cell sample was then loaded into the proposed ODEP microfluidic chip (Figure 1A) and succeeded by the two-step ODEP cell manipulation process (Figures 2A,B) (flow rate: 1.0 μl min^–1^, angle between the rectangular light bar and flow direction of the cell suspension: 15°) to separate and then isolate the SW620 cancer cells from magnetic microbead-bound Jurkat cells. In each step of cell manipulation process (i.e., Figures 2A,B), the cell purity of SW620 cancer cells in the harvested cell sample was evaluated with the aid of immunofluorescence microscopic observation. In the evaluation, the cell purity (%) of SW620 cancer cells was defined as the number of SW620 cancer cells isolated/the total cell number in the obtained cell sample^∗^100%.

## Results and Discussion

### The Optimal Operating Conditions for the Separation of SW620 Cancer Cells and Magnetic Microbead-Bound Jurkat Cells via ODEP

**FIGURE 3:**
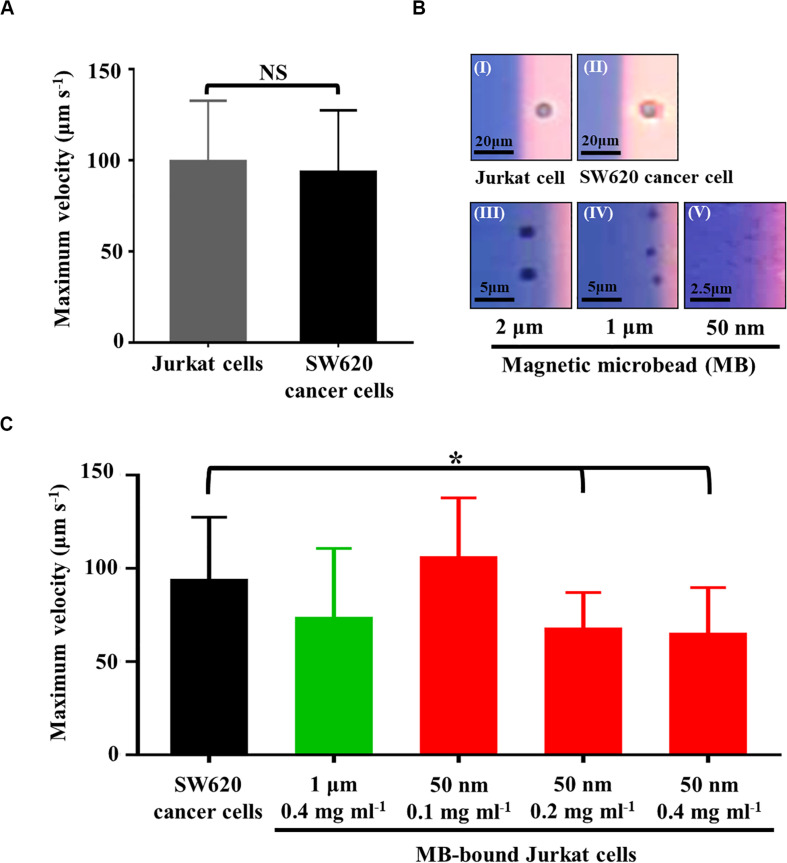
**(A)** comparison of the maximum velocity of a moving light bar that can manipulate SW620 cancer cells and Jurkat cells, **(B)** the microscopic images showing the (I) Jurkat cell and (II) SW620 cancer cell were attracted within the light image, and the (III) 2 μm, (IV) 1 μm, and (V) 50 nm magnetic microbead (MB) were repulsed and thus located outside of the light image, **(C)** comparison of the maximum velocity of a moving light bar that can manipulate the SW620 cancer cells and the MB-bound Jurkat cells with different conditions as indicated [Data were presented as mean ± standard deviation (*n* > 3). One-way ANOVA and the Tukey honestly significant difference (HSD) *post hoc* test were used for the statistical analysis. NS, No Significant difference (*p*>0.05), ^∗^Significant difference (*p*<0.05)].

In this study, ODEP cell manipulation (Figures 2A,B) was designed to process the cell samples obtained from a negative selection-based CTC isolation process. Owing to the heterogeneous property of CTCs, certain CTCs and leukocytes are similar in size to their model cells (i.e., SW620 cancer cells and Jurkat cells, respectively) used in this study (Hyun and Jung, 2014). In this situation, they might not be sorted and separated by the ODEP force because cellular size plays an important role in the ODEP force generated on these cells based on Eq. 1. This issue was further confirmed by Figure 3A, showing that the measured maximum velocities of a moving light bar (and thus the ODEP manipulation force) that can manipulate the SW620 cancer cells and Jurkat cells had no significant difference (*p* > 0.05). However, leukocytes are normally connected with magnetic microbeads after a negative selection-based CTC isolation process (Chiu et al., 2016; Liao et al., 2017, 2018; Kang et al., 2019). As discussed earlier, the nature of the ODEP force generated on a microparticle is dependent on the dielectric property of the microparticle at a given frequency of electric voltage (Chiou et al., 2005; Valley et al., 2008; Hwang and Park, 2011; Chen and Yuan, 2019). It was reported that microbeads and biological cells might exhibit negative and positive ODEP forces, respectively, under specific electric conditions (Chiou et al., 2005; Hwang and Park, 2011; Chen and Yuan, 2019). At a given electric condition of 10 Vpp and 3 MHz, this outcome was also experimentally confirmed by Figure 3B, in which the Jurkat cell and SW620 cancer cell were all attracted within the light image, whereas the magnetic microbeads with varied diameters were repulsed by the light image and thus located outside of the light image. Based on this fact, we speculated that the ODEP force generated on the magnetic microbead-bound leukocytes could be downregulated in comparison with the native leukocytes or SW620 cancer cells. If this is the case, the magnetic microbead-bound leukocytes and CTCs can then be further separated using ODEP after a conventional negative selection-based CTC isolation process.

To test this speculation and to determine the optimal operating conditions of magnetic microbeads (e.g., size and concentration) for separating magnetic microbead-bound Jurkat cells and SW620 cancer cells, experimental evaluations were carried out. In this study, Jurkat cells were bound with streptavidin-coated magnetic microbeads of different sizes (diameter: 2, 1 μm, and 50 nm) and different concentrations (e.g., 0.1, 0.2, and 0.4 mg ml^–1^) via the aid of biotin-coated anti-human CD45 antibodies. The ODEP manipulation force generated on the magnetic microbead-bound Jurkat cells and SW620 cancer cells was then evaluated experimentally. Within the experimental conditions explored, the results (Supplementary Figuer 2A) revealed that the use of a larger magnetic microbead (i.e., the 2 μm microbeads) could not lead to a significant difference (*p* > 0.05) in the maximum velocities of a moving light bar that can manipulate the magnetic microbead-bound Jurkat cells and SW620 cancer cells. For the use of magnetic microbeads slightly smaller than the previous microbead (i.e., the 1 μm microbeads), the results (Supplementary Figuer 2B) demonstrated that the maximum velocities of a moving light bar that can manipulate the magnetic microbead-bound Jurkat cells and SW620 cancer cells showed significant difference (*p*< 0.05) when the highest concentration (i.e., 0.4 mg ml^–1^) of magnetic microbeads was used. When the smallest magnetic microbeads (i.e., the 50 nm microbeads) were used, conversely, the maximum velocities of a moving light bar that can manipulate the magnetic microbead-bound Jurkat cells and SW620 cancer cells all exhibited significant differences (*p*< 0.05) within the concentration conditions tested (Supplementary Figuer 2C). For the 4 cases showing significant differences, overall, it was observed that the maximum velocities of a moving light bar that can manipulate the magnetic microbead-bound Jurkat cells were significantly lower than those of SW620 cancer cells. These findings, to some extent, justified our speculation that the ODEP force generated on a cell could be fine tuned if microparticles with a negative ODEP force nature (e.g., magnetic microbeads) are bound to it. This phenomenon is valuable for the combination of immunomagnetic microbead-based techniques and ODEP for cell separation and isolation. Moreover, it was also observed that the binding of cells with smaller magnetic microbeads might easily downregulate the ODEP manipulation force on cells (Supplementary Figuer 2). This finding could be due to the steric barrier effect, by which the quantity of larger magnetic microbeads binding on a cell is less than that of the smaller counterpart (Chen and Park, 2018). Based on the evaluations mentioned above, the operating conditions that lead to a significant difference in the ODEP manipulation force between the magnetic microbead-bound Jurkat cells and SW620 cancer cells were then selected for the following tests.

In the subsequent evaluations, the magnetic microbeads with the selected operating conditions (i.e., 1 μm magnetic microbeads with concentration of 0.4 mg ml^–1^ and 50 nm magnetic microbeads with concentrations of 0.1, 0.2, and 0.4 mg ml^–1^) were further tested by a real application [i.e., the negative selection-based cancer cell isolation process using the standard immunomagnetic microbeads-based cell isolation technique (Chiu et al., 2016; Liao et al., 2017, 2018; Kang et al., 2019)]. In the tests, the magnetic microbead-bound Jurkat cells remaining in the sample obtained after a negative selection-based cancer cell isolation process were harvested. The maximum velocities of a moving light bar that can manipulate these magnetic microbead-bound Jurkat cells were then measured and compared to those of SW620 cancer cells. The key purpose of this test was to examine whether the ODEP manipulation force acting on these magnetic microbead-bound Jurkat cells was similar to the results described earlier (Supplementary Figuer 2). The results (Figure 3C) showed that the maximum velocities of a moving light bar that can manipulate the magnetic microbead-bound Jurkat cells remaining in the samples obtained from the negative selection-based cancer cell isolation process (using 50 nm magnetic microbeads at concentrations of 0.2 and 0.4 mg ml^–1^) were significantly lower (*p*< 0.05) than those of SW620 cancer cells. This result was in line with that in the preliminary test (Supplementary Figuer 2). Nevertheless, the other two operating conditions (i.e., 1 μm microbeads at 0.4 mg ml^–1^ and 50 nm microbeads at 0.1 mg ml^–1^) demonstrated no significant difference (*p* > 0.05) in the maximum velocities of a moving light bar that can manipulate the magnetic microbead-bound Jurkat cells and SW620 cancer cells. The inconsistency between the result found here (Figure 3C) and that in the preliminary test (Supplementary Figuer 2) could be due to the selection effect of the magnetic field exerted during the negative selection-based cancer cell isolation process. Through this effect, most of the Jurkat cells with more magnetic microbead binding were attracted by the magnetic field and then removed in the subsequent process, leaving the Jurkat cells with less magnetic microbead binding remaining in the sample. Therefore, these Jurkat cells with less magnetic microbead binding showed no significant difference in ODEP manipulation force in comparison with SW620 cancer cells (Figure 3C). Based on the evaluations described above, overall, 50 nm magnetic microbeads at a concentration of 0.2 mg ml^–1^ were determined for the following works. Under the chosen condition, the maximum velocities of a moving light bar that can manipulate the magnetic microbead-bound Jurkat cells and SW620 cancer cells were 67.2 ± 19.9 and 93.3 ± 34.1 μm s^–1^, respectively (Figure 3C).

### The Optimal Operating Conditions for Isolating SW620 Cancer Cells From Magnetic Microbead-Bound Jurkat Cells Based on the Proposed Method

**FIGURE 4:**
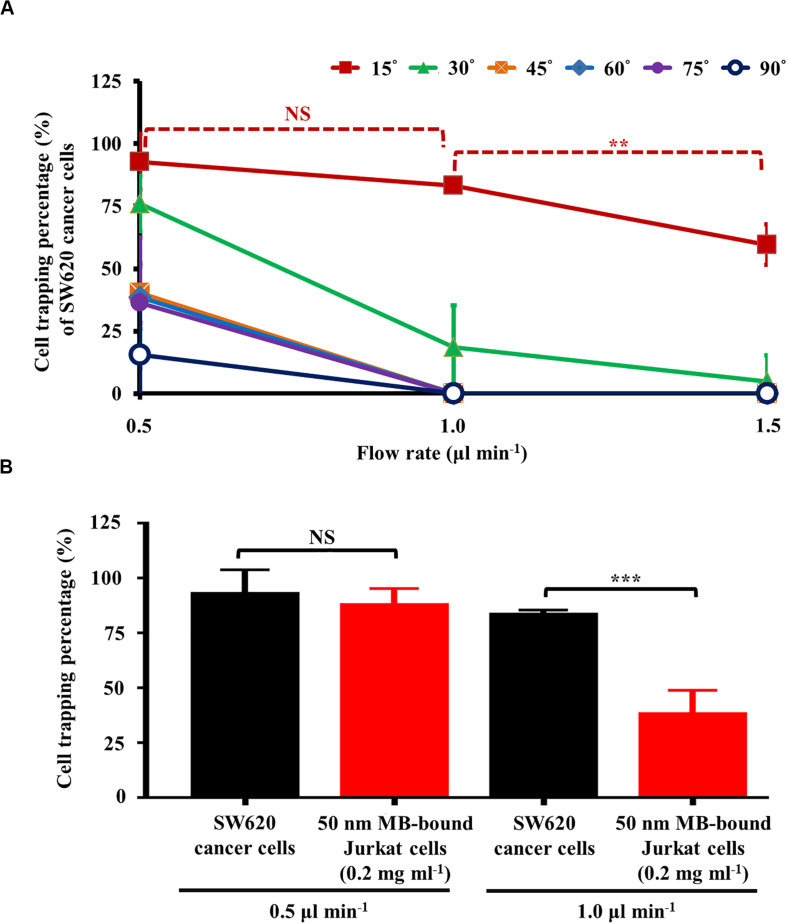
**(A)** the cell trapping percentage (%) of SW620 cancer cells trapped at the rectangular light bar area under different flow rates (0.5, 1.0, and 1.5 μl min^–1^) and angles (i.e., the angle between the light bar image and the flow direction of the sample flow) (15°, 30°, 45°, 60°, 75°, and 90°), and **(B)** comparison of cell trapping percentage (%) between SW620 cancer cells and 50 nm magnetic microbead (MB)-bound Jurkat cells (concentration: 0.2 mg ml^–1^) under the selected flow rates (i.e., 0.5 and 1.0 μl min^–1^, set angle: 15°) [Data were presented as mean ± standard deviation (*n* > 3). One-way ANOVA and the Tukey honestly significant difference (HSD) *post hoc* test were used for the statistical analysis. NS, No Significant difference (*p*>0.05), ^∗∗^Significant difference (*p*<0.01), ^∗∗∗^Significant difference (*p*<0.001)].

As discussed earlier, a static rectangular light bar with a particular angle to the flow direction of the cell suspension was designed in this study (Figure 2AI). As described in Figure 2C, the angle (between the light bar image and the direction of the sample flow), as well as the flow rate of the cell sample flow (thus the hydrodynamic force on cells; Eq. 2), play roles in the cell sorting and separation performance. To determine the optimal operating conditions, an experimental evaluation was carried out. In this work, the percentage (%) of SW620 cancer cells and the magnetic microbead-bound Jurkat cells trapped at the rectangular light bar area were experimentally evaluated under different angles (15°, 30°, 45°, 60°, 75°, and 90°) and flow rates (0.5, 1.0, and 1.5 μl min^–1^). Except for the angle condition of 15°, the results (Figure 4A) showed that the cell trapping percentage (%) of SW620 cancer cells decreased significantly with an increase in flow rate from 0.5 to 1.5 μl min^–1^. For a given flow rate condition, moreover, the increase of angle might lead to a dramatic downregulation in the cell trapping percentage (%) of cancer cells particularly when the flow rate was higher than 1.0 μl min^–1^. This finding could be explained by the fact that an increase in angle or flow rate (and thus F_HD_; Eq. 2) might accordingly lead to an increase in the hydrodynamic force component (i.e., F_HD_^∗^sinΘ) (Figure 2C). When the F_HD_^∗^sinΘ was greater than the F_ODEP_ acting on a cell, the cell flowing through the light image area might not be trapped by the light image (Figure 2CII). Based on the above evaluation, the optimal angle was determined to be 15°, for which the cell trapping percentages (%) of cancer cells were 92.8 ± 11.0, 83.3 ± 2.2, and 59.6 ± 8.2% under flow rate conditions of 0.5, 1.0, and 1.5 μl min^–1^, respectively. Because the cell trapping percentages (%) of cancer cells (i.e., 92.8 ± 11.0 and 83.3 ± 2.2%) showed no significant difference (*p*>0.05) under the flow rate conditions of 0.5 and 1.0 μl min^–1^ (set angle: 15°), the two operating conditions were selected for the following experimental evaluation.

**FIGURE 5:**
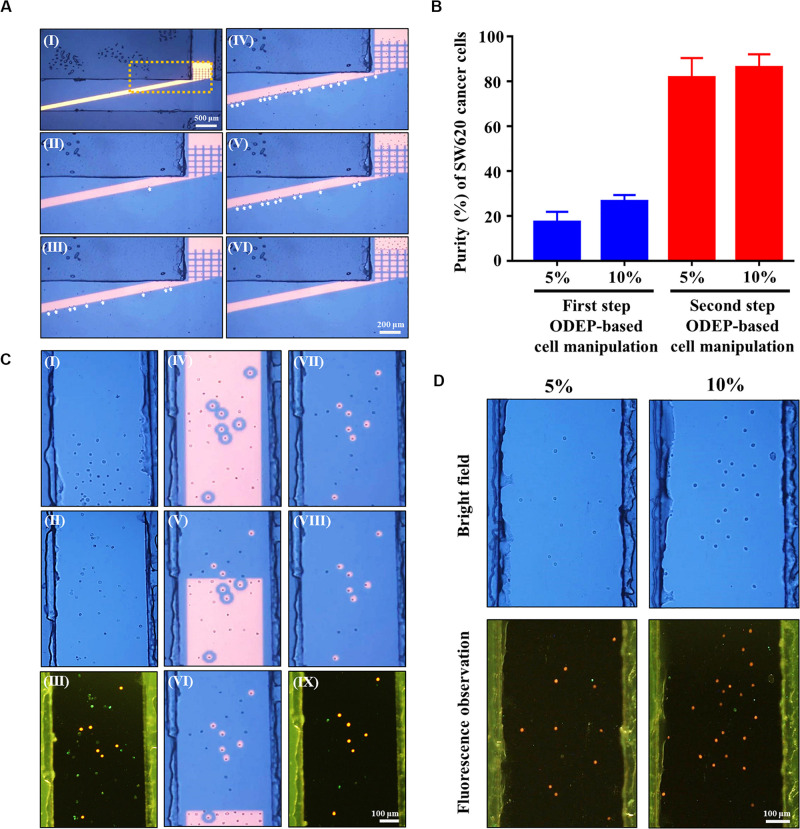
**(A)** microscopic observations of the first-step ODEP cell manipulation process; (I) a static rectangular light bar (L: 3.9 mm, W: 100 μm) with a particular angle (e.g., 15°) to the flow direction of the cell suspension was designed in the main microchannel, (II–VI) the SW620 cancer cells (indicated by arrows) were sorted, separated, and then guided along the rectangular light bar image to the side microchannel for collection (a video clip is provided as Supplementary Videos 1, 2), **(B)** the purity (%) of SW620 cancer cells obtained after the first and second step ODEP cell manipulation processes for the prepared cell samples originally containing 5 and 10% SW620 cancer cells, respectively, **(C)** microscopic observations of the second-step ODEP cell manipulation process; (I) a certain amount of the cancer cells were collected in the side microchannel, (II) the liquid flow in the side microchannel was driven to flux the cells collected so that they were evenly spread within the side microchannel, (III) fluorescence microscopy observation was carried out to identify the species of the cells [i.e., fluorescence stained SW620 cancer cells (the red dots) and immunofluorescence stained magnetic microbead-bound Jurkat cells (the green dots)] collected in the side microchannel for positioning the SW620 cancer cells, (IV) static circular light images were illuminated on each SW620 cancer cell. Meanwhile, the side microchannel was illuminated with a rectangular light bar to manipulate the magnetic microbead-bound Jurkat cells into the side microchannel, in which O-ring-like non-illuminated patterns were designed as partitions to separate the light-illuminated SW620 cancer cells and the other cells, (V–VII) the rectangular light bar on the side microchannel was then moved to manipulate the magnetic microbead-bound Jurkat cells to remove them from the side microchannel, and this process was repeated for 5 times, (VII–IX) through the previous process, the cell purity of the SW620 cancer cells in the side microchannel was greatly improved (a video clip is provided as Supplementary Video 3), **(D)** microscopy observation of the cells collected in the side microchannel after two-step ODEP manipulation processes for the prepared cell samples originally containing 5 and 10% SW620 cancer cells, respectively (Bright field: the upper row, Fluorescence observation: the lower row, SW620 cancer cells: the red dots; 50 nm magnetic microbead-bound Jurkat cells: the green dots).

Moreover, in this work, the comparison of the cell trapping percentage between SW620 cancer cells and magnetic microbead-bound Jurkat cells under the selected flow rate conditions (i.e., 0.5 and 1.0 μl min^–1^, set angle: 15°) was performed to determine the optimal flow rate condition under which the two cell species could be adequately separated by the proposed method. The results (Figure 4B) revealed that the cell trapping percentages of the two cells tested showed no significant difference (*p*>0.05) when the flow rate was set at 0.5 μl min^–1^. Conversely, the cell trapping percentages (i.e., 83.3 ± 2.2 and 38.0 ± 11.0%; the SW620 cancer cells and magnetic microbead-bound Jurkat cells, respectively) of the two cell species explored exhibited to have significant difference (*p* < 0.05) when the higher flow rate condition (i.e., 1.0 μl min^–1^) was adopted. These findings are explained herein. As described in Figure 3C, the maximum velocities of a moving light bar that can manipulate the 50 nm magnetic microbead-bound Jurkat cells (concentration: 0.2 mg ml^–1^) and SW620 cancer cells (and thus the F_ODEP_ generated on them; Eq. 2) were significantly different. However, due to the use of a lower flow rate of 0.5 μl min^–1^, the hydrodynamic force components (i.e., F_HD_^∗^sinΘ) of the two cells tested could all be lower than their F_ODEP_ values (Figure 2CI). This outcome in turn led to the phenomenon that most of the cells flowing through the light image area were trapped by the designed light image. Therefore, the two cell species tested might not be adequately sorted and separated by the set operating conditions. Conversely, this phenomenon was improved greatly when the flow rate was increased to 1.0 μl min^–1^, by which the F_HD_^∗^sinΘ of the magnetic microbead-bound Jurkat cells and SW620 cancer cells could be higher (Figure 2CII) and lower (Figure 2CI) than their F_ODEP_ values, respectively. In this situation, the two cell species explored could be adequately sorted and separated by the proposed method. Based on the evaluations (Figure 4), overall, the angle (between the light bar image and the flow direction of sample flow) and the flow rate of the cell sample were set at 15° and 1.0 μl min^–1^, respectively. For the latter, particularly, the flow rate (1.0 μl min^–1^) used in this work was significantly higher than that [e.g., 0.1 (Huang et al., 2013) and 0.4 μl min^–1^ (Chou et al., 2017)] in the other ODEP-based microfluidic system for cell sorting, separation, or isolation. This outcome might, to some extent, address the technical problem of the low working throughput for ODEP microfluidic systems for similar applications (Huang et al., 2013; Chou et al., 2017).

### Performance Evaluation of SW620 Cancer Cell Isolation Using the Proposed Method

In this study, the performance of using the proposed method (Figure 2) for refining the cell purity of cancer cells obtained after a conventional negative selection-based CTC isolation scheme (Chiu et al., 2016; Liao et al., 2017, 2018; Kang et al., 2019) was experimentally evaluated. Because the CTC purity in the cell samples obtained from a conventional negative selection-based CTC isolation scheme is normally in the range of 5~10% (Liao et al., 2017; Kang et al., 2019), the cell samples containing 5 and 10% SW620 cancer cells were then prepared to mimic the cell samples obtained after the first-step CTC isolation process in this work. The prepared cell sample was then loaded into the proposed ODEP microfluidic chip (Figure 1A), followed by the two-step ODEP cell manipulation process (Figures 2A,B) (flow rate: 1.0 μl min^–1^, angle: 15°) to separate and then isolate SW620 cancer cells from the 50 nm magnetic microbead-bound Jurkat cells. Figure 5A shows the microscopic observations of the first-step ODEP cell manipulation process (Figure 2A), in which the SW620 cancer cells (indicated by arrows) were sorted, separated, and then guided along the rectangular light bar image to the side microchannel for collection (a video clip is provided as Supplementary Videos 1, 2). In this work, the purities of cancer cells collected in the side microchannel were evaluated to be 17.2 ± 4.6 and 26.4 ± 2.9%, respectively, for the prepared cell samples originally containing 5 and 10% SW620 cancer cells (Figure 5B). After the first step of the ODEP cell manipulation process, overall, the cell purity of cancer cells might be improved by approximate 2.6~3.4-folds increase (Figure 5B). After the initial continuous cell isolation and purification process (Figure 5A), the second step of the ODEP cell manipulation was carried out in the side microchannel to further refine the cell purity of cancer cells by removing the 50 nm magnetic microbead-bound Jurkat cells existing in the cell sample, as shown in (Figure 5C) (a video clip is provided as Supplementary Video 3). Through this process, it was found that most of the magnetic microbead-bound Jurkat cells were successfully removed, leaving the isolated cancer cells with the cell purity as high as 81.6 ± 8.8 and 86.1 ± 6.0%, respectively, for the prepared cell samples originally containing 5 and 10% SW620 cancer cells (Figure 5B). Microscopy-observed results were shown in Figure 5D. When a viability dye (CellTrace^TM^ Calcein Red-Orange) was used to stain the SW620 cancer cells, it can be observed that almost all of the isolated cancer cells were viable (Figure 5D). Furthermore, the number of SW620 cancer cells that could be isolated at a given amount of time was experimentally evaluated to be 7.3 ± 1.5 cells per 10 min and 20.3 ± 4.3 cells per 10 min for mixed samples containing 5 and 10% SW620 cancer cells, respectively. Overall, the experimental evaluation has demonstrated the feasibility of using the immunomagnetic bead-based cell isolation and ODEP cell manipulation for the negative selection-based isolation of cancer cells.

### Technical Features of Using the Presented Method for the High Purity Isolation of Cancer Cells After a Negative Selection-Based CTC Isolation Process

CTC isolation based on a negative selection-based strategy is believed to be able to harvest all possible cancer cells in the blood samples of cancer patients (Chiu et al., 2016; Gabriel et al., 2016; Peters et al., 2016; Liao et al., 2017, 2018; Kang et al., 2019). The viable, label-free, and particularly physiologically meaningful cancer cells harvested via the method are valuable clinically (Chiu et al., 2016; Gabriel et al., 2016; Liao et al., 2017, 2018; Kang et al., 2019). However, its main technical shortcoming is its inability to isolate high-purity cancer cells, making the subsequent analytical work complicated and technically demanding. To tackle this technical hurdle, the utilization of ODEP-based cell manipulation in a simple microfluidic system was presented to process the cell samples obtained from a negative selection-based CTC isolation process to refine the cell purity of cancer cells. In the cell sample obtained from a negative selection-based CTC isolation process, the predominant cells in the sample are leukocytes and few (e.g., 5~10%; Liao et al., 2017; Kang et al., 2019) of them are the cell population containing conventionally defined EpCAM^+^ CTCs or EMT-transformed cancer cells (Chiu et al., 2016; Gabriel et al., 2016; Liao et al., 2017, 2018; Kang et al., 2019). Because the two cell populations (i.e., leukocytes and cancer cells) in the sample have similar cellular sizes (Chen et al., 2014; Hyun and Jung, 2014; Hao et al., 2018), it makes them technically difficult to separate by the size-based cell separation methods (Chen et al., 2014; Hyun and Jung, 2014; Hao et al., 2018), including the ODEP-based approach (Chen et al., 2014). To address this issue, one of the characteristic features of the proposed method is the combination of immunomagnetic microbead-based techniques and ODEP for cell separation and isolation. In the negative selection-based CTC isolation process, the immunomagnetic microbead-based technique allows the leukocytes to be labeled with magnetic microbeads. Because the nature of the ODEP force generated on a microparticle is dependent on the dielectric property of the microparticle (Chiou et al., 2005; Valley et al., 2008; Hwang and Park, 2011; Chen and Yuan, 2019), the binding of magnetic microbeads on the leukocytes could alter their dielectric properties. In this situation, ODEP cell manipulation could be used to separate the magnetic microbead-bound leukocytes from the cell population containing cancer cells.

For efficient and high-purity cancer cell isolation, the other technical feature of the proposed method was the design of a two-step ODEP cell manipulation process, schematically illustrated in Figures 2A,B. In the process, ODEP virtual cell filter and track were first used to initially sort and separate cancer cells and then isolate them from the surrounding magnetic microbead-bound leukocytes in a continuous flow manner. This step was followed by immunofluorescence microscopic observation and ODEP cell manipulation to refine the cell purity of the harvested cancer cells. Through the two-step ODEP cell manipulation process, the presented method could largely improve the efficiency and cell purity of a CTC isolation process compared with other ODEP-based CTC isolation schemes (Huang et al., 2013).

Furthermore, other technical advantage of the proposed method is the design of a static rectangular light bar with a particular angle to the flow direction of the cell suspension at the defined cell isolation zone of the main microchannel (Figure 2AI). The designed light bar functioned both as a virtual cell filter and as a virtual cell track that simultaneously sorted, separated, and guided the cells to be isolated to the side microchannel for collection. This design not only largely simplifies the design of light images for cell manipulation but also contributes to a higher working throughput in comparison with the other ODEP-based continuous CTC isolation schemes (Huang et al., 2013; Chou et al., 2017). In the ODEP microfluidic system for cell sorting and separation (Chou et al., 2017), for example, static light images are normally designed to be perpendicular to the direction of a cell sample flow. For the cells to be isolated, the hydrodynamic force acting on the cells is normally set to be lower than the ODEP manipulation force of desirable cells to trap them within the light images. In this situation, the set hydrodynamic force on cells and thus the working flow rate of the cell suspension in a microfluidic system is limited (Eq. 2) because the ODEP manipulation force of cells is generally weak. Compared to the conventional design, a static rectangular light bar with a particular angle (Θ) to the flow direction of the cell suspension was designed in this work. For the cells to be isolated (e.g., SW620 cancer cells), the component of hydrodynamic force (i.e., hydrodynamic force^∗^sinΘ) was designed to be lower than the ODEP manipulation force of desirable cells to trap the cells within the light images (Figure 2CI). In this situation, the set hydrodynamic force on cells (and thus the working flow rate of the cell suspension; Eq. 2) could be higher than that of the conventional case (Huang et al., 2013; Chou et al., 2017). In other words, the working flow rate of sample flow in the presented design can be higher than that in the conventional case, which could accordingly contribute to a higher working throughput. This design might, to some extent, address the technical problem of the low working throughput of ODEP-based microfluidic systems for cell sorting, separation, or isolation. Due to the technical limitation (i.e., experimental setup), moreover, the minimum angle of the rectangular light bar to the flow direction was set at 15° in this study. In future work, it might be worthy investigating if the cell trapping percentage (%) could be improved when the angle is lower than 15°.

## Conclusion

The negative selection-based CTC isolation scheme features the capability to harvest viable, label-free CTCs, particularly all possible CTCs, including conventionally defined EpCAM^+^ CTCs and EMT-transformed cancer cells, from the blood samples of cancer patients. The physiologically meaningful cancer cells harvested via this method are valuable clinically. Nevertheless, its key technical shortcoming is its inability to isolate CTCs in a high purity manner, which could limit the subsequent use of the obtained CTCs for analytical work. To address this issue, this study proposed a two-step ODEP cell manipulation to process the cell sample harvested from a negative selection-based CTC isolation process. In operations, an ODEP-based virtual cell filter and track were first used to initially sort, separate, and then isolate the cancer cells from the surrounding magnetic microbead-bound cells in a continuous manner. This step was followed by immunofluorescence microscopic observation and ODEP cell manipulation to refine the purity of the harvested cancer cells. Through the two-step ODEP cell manipulation process, the presented method could improve the working throughput and cell purity of a CTC isolation process compared with other ODEP-based CTC isolation schemes. In this study, an ODEP microfluidic system was designed and fabricated. The optimum ODEP operating conditions [e.g., size (50 nm) and concentration (0.2 mg ml^–1^) of magnetic microbeads, the angle between the rectangular light bar and sample flow direction (15°), and the sample flow rate (1.0 μl min^–1^)] for effective cell separation and isolation were determined experimentally. The results revealed that the presented two-step ODEP cell manipulation process was able to further refine the cancer cell purity of the sample obtained after a negative selection-based CTC isolation process with high cell purity (81.6~86.1%). In this proof-of-concept study, we have used cell lines as a model to develop the technique. In our future work, we will expand our studies into clinical samples (i.e., the isolation and purification of CTCs from patient samples of different cancer types) to justify its clinical validity. Although the combination of immunomagnetic bead-based cell isolation and ODEP cell manipulation for the negative selection-based isolation of CTCs has been successfully demonstrated, the technique developed in this study is still in its early stage of development. Further system integration, including automation, would greatly enhance the utilization of this technique to perform isolate and purify large number of label-free and pure rare cells in biological samples.

## Data Availability Statement

All datasets presented in this study are included in the article/Supplementary Material.

## Author Contributions

P-YC, C-HH, and M-HW conceived and designed the experiments and drafted the manuscript. P-YC and C-HH conducted experiments and conducted data analysis. All authors contributed to the article and approved the submitted version.

## Conflict of Interest

The authors declare that the research was conducted in the absence of any commercial or financial relationships that could be construed as a potential conflict of interest.
