# Supplementary material for: The Combination of Immunomagnetic Bead-Based Cell Isolation and Optically Induced Dielectrophoresis (ODEP)-Based Microfluidic Device for the Negative Selection-Based Isolation of Circulating Tumor Cells (CTCs)
Source: Front Bioeng Biotechnol. 2020 Aug 6;8:921. doi: 10.3389/fbioe.2020.00921 (PMC7438881; doi:10.3389/fbioe.2020.00921)
Supplement: Supplementary file 1 [file Data_Sheet_1.PDF]

Supplementary Material

1 Supplementary Figures

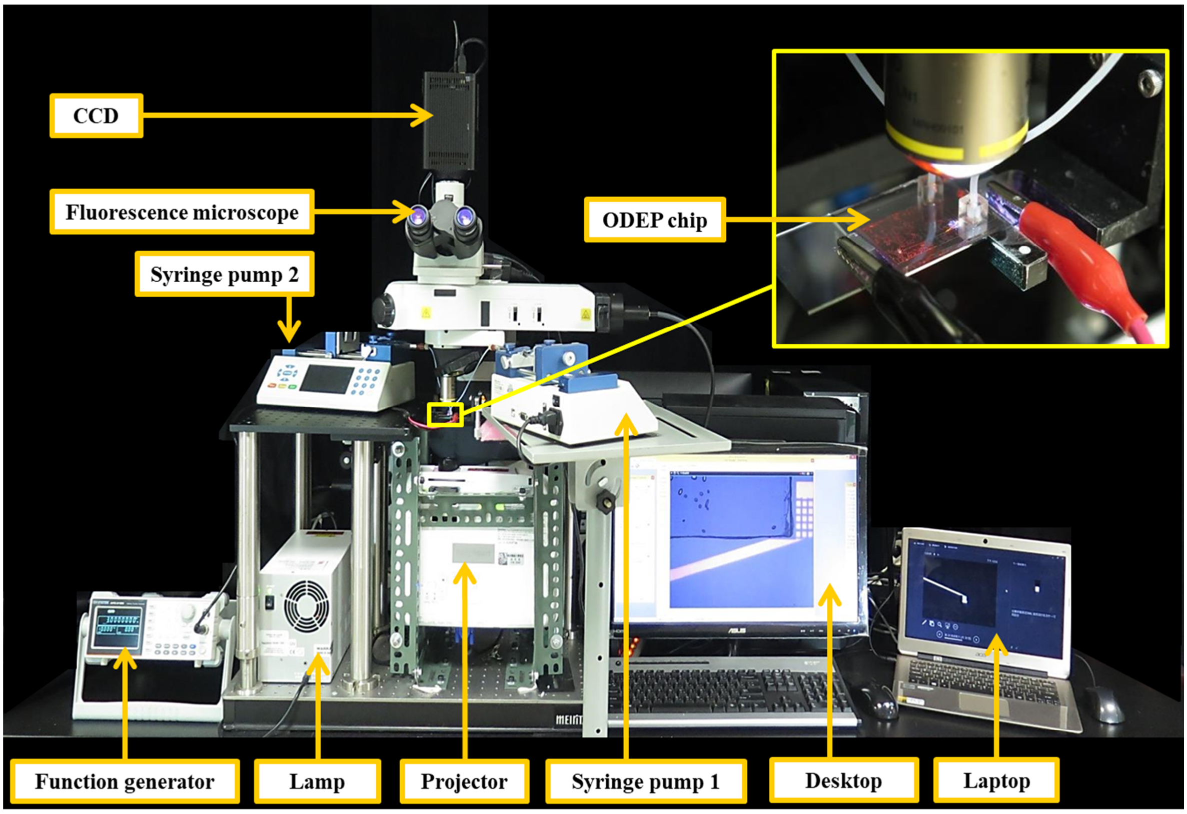

Supplementary Figure 1. Photograph of the whole operating setup

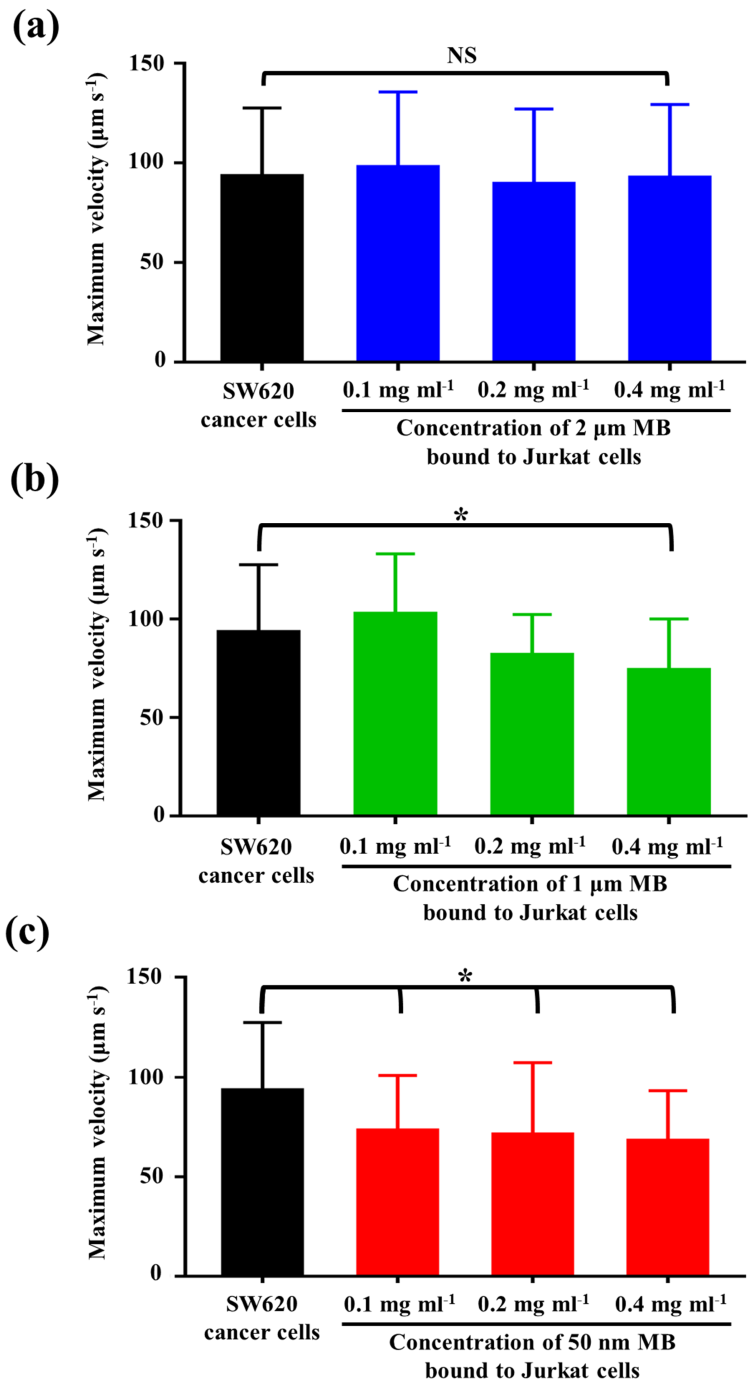

Supplementary Figure. 2 Comparison of the maximum velocity of a moving light bar that can manipulate SW620 cancer cells and Jurkat cells bound with the **(a)** 2 µm, **(b)** 1 µm, and **(c)** 50 nm magnetic microbead (MB) with varied concentrations (0.1, 0.2, and 0.4 mg ml^{-1}) [Data were presented as mean ± standard deviation (n>3). One-way ANOVA and the Tukey honestly significant difference (HSD) post hoc test were used for the statistical analysis. NS: No Significant difference ($p > 0.05$), * Significant difference ($p < 0.05$)].

2 Supplementary Videos

Video Clip 1: The first-step ODEP cell manipulation process for continuous cancer cell isolation; the overview

Video Clip 2: The first-step ODEP cell manipulation process for continuous cancer cell isolation; the close-up view

Video Clip 3: The second-step ODEP cell manipulation process for cancer cell isolation and purification
